# Room Temperature Magnetic Memory Effect in Cluster-Glassy Fe-Doped NiO Nanoparticles

**DOI:** 10.3390/nano10071318

**Published:** 2020-07-04

**Authors:** Ashish Chhaganlal Gandhi, Tai-Yue Li, B. Vijaya Kumar, P. Muralidhar Reddy, Jen-Chih Peng, Chun-Ming Wu, Sheng Yun Wu

**Affiliations:** 1 Department of Physics, National Dong Hwa University, Hualien 97401, Taiwan; acg.gandhi@gmail.com (A.C.G.); tim312508@gmail.com (T.-Y.L.); 2 Department of Chemistry, Nizam College, Osmania University, Hyderabad 500001, India; vijaychemou@gmail.com; 3 Department of Chemistry, University College of Science, Osmania University, Hyderabad 500007, Telangana, India; pmdreddy@gmail.com; 4 SIKA, National Synchrotron Radiation Research Center, Hsinchu 30076, Taiwan; peng.hanz@nsrrc.org.tw (J.-C.P.); cmw@ansto.gov.au (C.-M.W.)

**Keywords:** room temperature, magnetic memory effect, intraparticle interactions, 4:1 defect cluster, Fe-doped NiO

## Abstract

The Fe-doped NiO nanoparticles that were synthesized using a co-precipitation method are characterized by enhanced room-temperature ferromagnetic property evident from magnetic measurements. Neutron powder diffraction experiments suggested an increment of the magnetic moment of 3*d* ions in the nanoparticles as a function of Fe-concentration. The temperature, time, and field-dependent magnetization measurements show that the effect of Fe-doping in NiO has enhanced the intraparticle interactions due to formed defect clusters. The intraparticle interactions are proposed to bring additional magnetic anisotropy energy barriers that affect the overall magnetic moment relaxation process and emerging as room temperature magnetic memory. The outcome of this study is attractive for the future development of the room temperature ferromagnetic oxide system to facilitate the integration of spintronic devices and understanding of their fundamental physics.

## 1. Introduction

The antiferromagnetic (AF) metal oxide nanoparticles (NPs) have attracted enormous attention because of their promising technological applications and fundamental physics. The effect of finite size leads to the accumulation of frustrating surface spins and various point defects at the surface of the AF NPs, which results in an interesting magnetic and optical properties that differ significantly from their bulk counterparts [1]. Among the various AF materials, nickel oxide (NiO) is one of the few *p*-type semiconductors (acceptor state induced by the nickel vacancy (V_Ni_) with a wide-bandgap *E_g_* = 4 eV) having face-centered-cubic (*fcc*) crystal symmetry. In the bulk form, NiO possesses AF ordering with the Neel transition temperature *T_N_* of 523 K [2]. However, NiO nanostructure exhibits anomalous magnetic properties that are very sensitive to size, Ni vacancy defects, morphology, and crystal structure, thus showing a wide variety of intriguing phenomena. It has been reported that below a particle size of *d* = 30 nm, the long-range ordered Ni^2+^─O^2-^─Ni^2+^ superexchange interaction breakdown, due to an enhanced V_Ni_ defect resulting in a weak ferromagnetic (FM) like properties [3,4]. Furthermore, the effect of frustrating surface spins become more dominant below *d* = 10 nm [5,6,7]. The NiO nanostructures have been used in rechargeable batteries [8], magnetic recording media [9], the next-generation resistive switching memory devices [10], and so on. Recently, functionalized NiO nanostructures have attracted great research interest from both fundamental and application point of view [11,12]. The further development in functionalized NiO nanostructure is focused on obtaining a room temperature (RT) ferromagnetism without comprising the structure in order to facilitate the possible integration of spintronic devices.

According to recent findings, RT ferromagnetism in NiO NPs can be achieved through transition metal (TM)-doping, for example, Fe (either due to substitution or the formed defect clusters), which opens up their potential applications in the future advanced spintronic devices [13,14,15]. The properties of such a system can be tailored by controlling both the particle size and Fe-dopant concentration, which results in a complex magnetic property [16,17,18,19,20,21,22]. For instance, the doping of Fe^3+^ ions in NiO either could replace the Ni ions or occupy an interstitial site. The substituted Fe^3+^ ions can alter the Ni^2+^─O^2−^─Fe^3+^ superexchange interaction and so the AF properties. Whereas, Fe ions at the interstitial site could form 4:1 defect cluster consisting of tetravalent interstitial ${\mathrm{Fe}}_{i}^{4+}$ and four V_Ni_, in total being four times negatively charged [16,23,24,25,26,27]. Such a complex structure could result in interesting magnetic properties. According to previous reports, bare and the Fe-doped NiO NPs both exhibit low-temperature magnetic memory effect [16,28,29]. Such a type of nanoscale system can be used as a “thermal assistant memory cell” in digital information storage [30,31,32].

However, the magnetic memory effect is mostly observed in the low-temperature region far below the RT, and this is the major obstruct precluding its application in nanotechnology. In the past, the above obstacle has been foiled through introducing exchange-coupling [30,33] and particle size distribution [34]. In this study, we have reported RT magnetic memory effect from Fe-doped NiO NPs synthesized using a co-precipitation method followed by thermal treatment in the air. A thorough investigation of structural and magnetic properties was carried out using synchrotron radiation powder X-ray diffraction (PXRD), neutron powder diffraction (NPD), and dc magnetometer. Our findings suggest that the RT magnetic memory effect in Fe-doped NiO NPs is mediated through intrinsic intraparticle interactions.

## 2. Materials and Methods

**Scheme 1:**
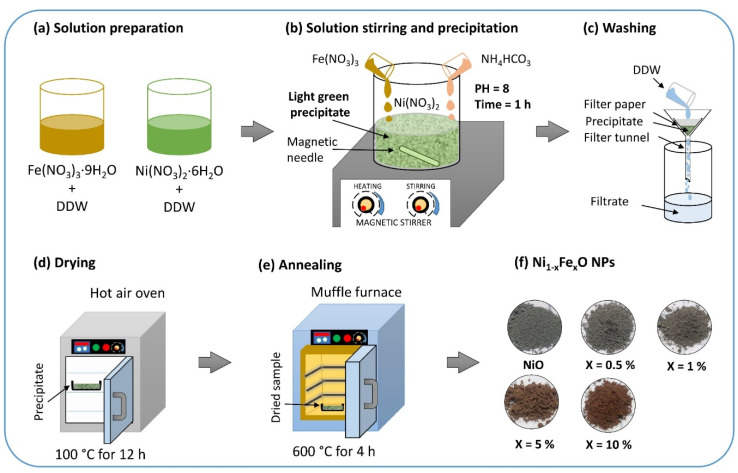
(**a**–**f**) Schematic representation of Ni_1-x_Fe_x_O NPs preparation process using the co-precipitation method.

All of the analytical grade Nickel (II) nitrate hexahydrate (Ni(NO_3_)_2_·6H_2_O), iron (III) nitrate nonahydrate (Fe(NO_3_)_3_·9H_2_O), and ammonium bicarbonate (NH_4_HCO_3_) were procured from S.D. Fine-Chemicals Ltd., India, and used as received without further purifications. NiO and Fe-doped Ni_1-x_Fe_x_O with x(%) varying from 0 to 10% compositions were prepared. A schematic Scheme 1 shows the process of Ni_1-x_Fe_x_O NPs preparation while using the co-precipitation method. The stoichiometric amounts of Ni(NO_3_)_2_ and Fe(NO_3_)_3_ (molar ratio of Ni to Fe is 0, 0.5, 1, 5, and 10%) were dissolved in double-distilled water (DDW) separately (Scheme 1a). Subsequently, ferric nitrate solution was added dropwise to nickel nitrate solution under continuous stirring for 1 h. The pH of the solution was maintained at 8 by adding the NH_4_HCO_3_ solution (Scheme 1b). The light green precipitate was formed by adding NH_4_HCO_3_. The resultant precipitate was washed several times with DDW (Scheme 1c) and then dried at 100 °C for 12 h in a hot air oven (Scheme 1d). The obtained powder was grounded in an agate mortar and calcined at 600 °C for 4 h in a muffle furnace (Scheme 1e). The obtained pure NiO powder was gray-green. The effect of Fe doping up to 1% leads to a slight change in color to light brownish gray-green. However, a drastic change in color to dark brown was noted on 5%, and on 10% powder becomes red-brown (Scheme 1d).

A morphological analysis of powdered samples was carried out using the field-emission scanning electron microscopy (FE-SEM, JEOL JSM-6500F microscope, Tokyo, Japan). Synchrotron radiation PXRD measurements were carried out at the National Synchrotron Radiation Research Center in Hsinchu, Taiwan (beamline BL01C2, λ = 0.7749 Å). For the investigation of magnetic structural properties, the unpolarised NPD spectra were collected on 2 g sample at 10 K and 300 K. The NPD experiments were carried out on SIKA (the high flux cold neutron triple-axis spectrometer) at OPAL reactor, ANSTO (λ = 2.35 Å). With a wide range of incident energy from 2.5 to 30 meV and three detector options provide great flexibility on neutron study. SIKA was configured as diffraction mode, which used diffraction detector to gain a strong diffraction signal. Incident energy was configured at 14.87 meV with open-open-60 collimation. Sample pre-slit and post-slit were adjusted to the sample size in order to improve the signal quality. Sample environment CF11 was used to control the sample temperature. The measurements were collected based on the counts of the beam monitor to ensure the precise neutron flux on the sample. The measurements of magnetic properties were carried out while using a superconducting quantum interference device (SQUID) magnetometer (Quantum Design, SQUID-VSM Ever Cool, San Diego, CA, USA).

## 3. Results

### 3.1. Morphological and Elemental Analysis

**Figure 1:**
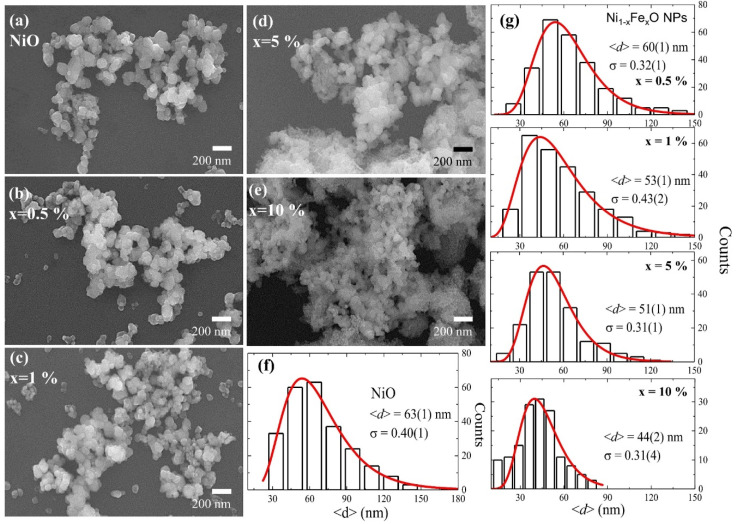
(**a**–**e**) SEM images of NiO, 0.5, 1, 5 and 10% samples, respectively. Histogram obtained from (**f**) NiO, (**g**) 0.5, 1, 5, and 10% samples (top to bottom). The solid red lines represent a fit using the Log-normal distribution function.

The facetted NPs with a broad shape distribution were observed from the SEM images of NiO, 0.5 to 10% samples that are shown in Figure 1a–e, respectively. From SEM images, an increase of aggregation of particles with the increase of doping concentration can be seen. The mean diameter <*d*> of NPs is estimated by fitting a log-normal distribution function: $f\left(d\right)=\frac{1}{\sqrt{2\pi }d\sigma }\exp\left[-\frac{\left(\ln d-\ln\langle d\rangle \right)}{2\sigma^{2}}\right]$ to the histogram that was obtained from SEM images of NiO (Figure 1f) and 0.5 to 10% sample (Figure 1g, top to bottom), where the value of *σ* represents a standard deviation of the fitted function. The effect of Fe-doping from 0 to 10% in NiO leads to a decrease of particle size (i.e., mean diameter <*d*>) from 63(1) nm to 44(2) nm. Furthermore, along with the big size particles, small size NPs with a diameter below <5 nm were also visible in SEM images of 5 and 10% samples (Figure 1d,e). The small size particles could be related to the Fe_3_O_4_ impurity phase (supported by an observed drastic color change and further confirmed from the synchrotron radiation PXRD experiment Figure S1 in the supporting information and the magnetic measurements) [32]. Fe_3_O_4_ is a ferrimagnetic material with a high Curie temperature (T_C_ = 850 K). The presence of such a strong magnetic impurity phase overshadows the intrinsic magnetic properties of Fe-doped NiO materials. The focus of the present study is to study the RT magnetic and memory effect from Fe-doped NiO NPs without compromising the structural properties. Hence, we will not discuss the 5% and 10% samples having the Fe_3_O_4_ impurity phase. The crystalline size that was obtained from the most intense (200) PXRD peak of 0 to 1% samples varies between 68 nm to 64 nm (Table S1). The decrease of particle size with the increase of Fe-concentration and appearance of small size NPs above 1% Fe-concentration are consistent with the previous findings [24].

### 3.2. Structural Properties

**Figure 2:**
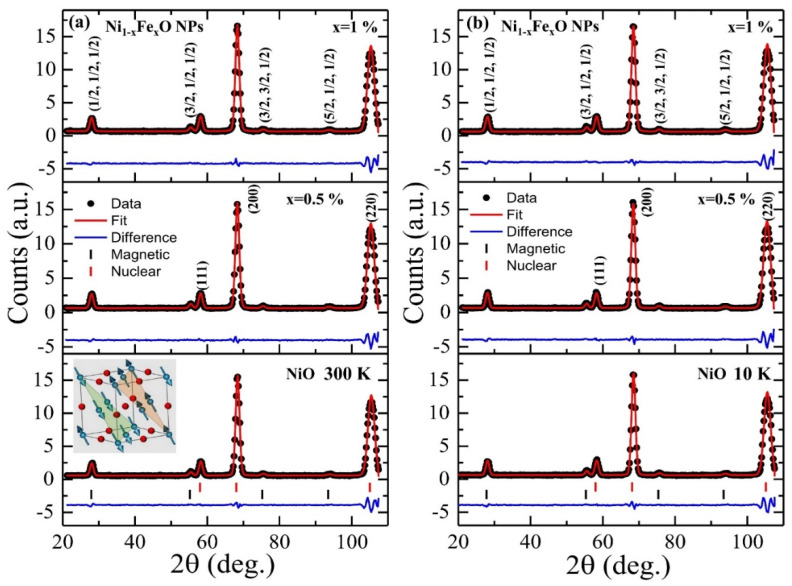
The Rietveld refined (red line) neutron powder diffraction (NPD) spectra (dots) from NiO, 0.5, and 1% samples (bottom to top) taken at (**a**) 300 K and (**b**) 10 K are calculated with $Fm\bar{3}m$ symmetry. The blue line represents the difference between experimental and fitted spectra. The vertical black and short red lines represent the magnetic and nuclear brags reflection positions. Inset in the bottom panel of (**a**): a unit cell of NiO with two magnetic sublattices.

In the paramagnetic phase (T > T_N_ = 523 K) NiO possesses cubic $Fm\bar{3}m$ symmetry. Below T_N_, the structure of NiO undergoes a weak cubic-to-rhombohedral distortion (space group *R*$\bar{3}m$) due to the magnetostriction effect. The rhombohedral distortion can simply be noticed from the splitting of (220) reflection in diffraction peak profile, and the effect increases with the decrease of temperature [35,36]. The NPD spectra of particles that were recorded at 300 K contain a peak originating from both the nuclear and magnetic origin (Figure 2). A slight enhancement in the intensity of the magnetic peak is noted on lowering the sample temperature to 10 K. However, we did not observe any splitting of (220) reflection in pure and Fe-doped NiO NPs. Therefore, initially, we have analyzed the diffraction in the cubic $Fm\bar{3}m$ symmetry with a propagation vector $k=\left(\frac{1}{2},\;\frac{1}{2},\;\frac{1}{2}\right)$ and a lattice parameter of 4.1783(2) Å. The Rietveld refined NPD spectra of NiO, 0.5%, and 1% samples taken at 300 K and 10 K are shown in Figure 2a,b (bottom to top), and Table S2 summarizes the corresponding fitting parameters. The refinement of the NPD spectra of NiO NPs corroborates the conventional two magnetic sublattices of NiO with antiparallel orientation within (111) plane (inset of Figure 2a, in the bottom panel). The large AF exchange interaction between the next-nearest-neighbor is at the origin of the Neel temperature of NiO [37]. At 300 K, the obtained value of the Ni ordered magnetic moment from NiO NPs is 1.232(18) $\mu_{B}$ and at 10 K, it enhances to 1.258(18) $\mu_{B}$ [37,38]. Rietveld refinement of NPD spectra was further carried out using rhombohedral *R*$\bar{3}m$ symmetry to examine whether pure and Fe-doped NiO NPs undergo cubic-to-rhombohedral distortion, and the corresponding fitting parameters are summarized in Table S3. Interestingly, an improved magnetic moment of 1.636(30)$\;\mu_{B}$ is obtained from NiO NPs at 300 K and 10 K, it enhances to 1.831(23)$\;\mu_{B}$, which is 8.45% lower than the spin-only value of 2.0${\;\mu }_{B}$. The obtained low value could be related to the presence of magnetically disordered shell at the surface of the NPs [39,40]. Note that, here, the overall goal of the NPD experiment is to understand the effect of Fe-doping on the magnetic moment of 3*d* ions in the AF NiO NPs. Hence, even though refinement using rhombohedral symmetry return results near to spin-only value than that of cubic symmetry, it will not affect the magnetic memory results as long as both symmetries yield a similar increase in magnetic moment trend with Fe-doping concentration. Furthermore, the observed difference between the results of the refinement is worth discussion; however, it is outside of the scope of current work and, therefore, can be discussed further in the near future. The subsequent analysis of NPD spectra from 0.5 and 1% samples using both cubic and rhombohedral symmetry yields a similar magnetic structure to that of undoped NiO NPs, but with an enhanced magnetic moment (see Tables S2 and S3). Therefore, no obvious change in the chemical structure was observed up to 1% Fe-doping, although the effect has led to an increment of the magnetic moment in the NPs.

### 3.3. Exchange Bias

**Figure 3:**
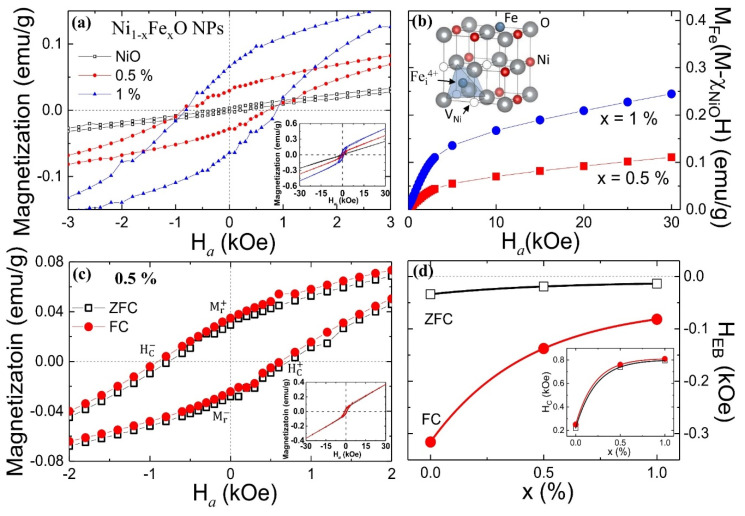
(**a**) Magnified ZFC-M(H*_a_*) loops near zero-field from Ni_1-x_Fe_x_O NPs at 300 K. (**b**) Field dependent magnetization M_Fe_(M-χ_NiO_H) from 0.5% and 1% samples. (**c**) Magnified ZFC-FC M(H*_a_*) loop measured at 300 K from 0.5% sample. (**d**) Composition x dependency of exchange-bias field H_EB_. Inset: (**a**) full ZFC-M(H*_a_*), (**b**) 4:1 defect cluster consisting of tetravalent interstitial ${\mathrm{Fe}}_{i}^{4+}$, four nickel vacancies V_Ni_, substituted Fe at Ni site (blue sphere), (**c**) full ZFC-FC M(H*_a_*) loops over ±30 kOe field, and (**d**) composition x-dependency of coercivity H_C_.

ZFC and FC magnetic hysteresis M(H*_a_*) loop measurements were both carried out at 300 K to study the effect of Fe-doping on the magnetic properties of NiO NPs. Initially, during ZFC measurement, the SQUID magnet was reset to remove any stray field at 400 K, followed by cooling the sample to the desired temperature. During FC measurement, the sample was cooled down from 400 K in an external magnetic field of 10 kOe to the desired temperature. Figure 3a shows the magnified ZFC M(H*_a_*) loops near zero-field, where inset gives full M(H*_a_*) loops measured over ±30 kOe from Ni_1-x_Fe_x_O NPs. The observed non-zero coercivity and the linear increasing behavior of the magnetization in the high-field region correspond to FM and AF two-component behavior, respectively. The value of coercivity (H_C_) and the magnetization increase with the increase of Fe-concentration from 0 to 1%. On the other hand, a saturation-like magnetization behavior with an enhanced magnetization is obtained from 5 and 10% samples attributed to the Fe_3_O_4_ impurity phase (Figure S2) [22]. The net enhancement in the magnetization because of Fe-doping $\left(M_{Fe}=M-\chi_{\mathrm{NiO}}H\right)$ can be quantified by subtracting the contribution from NiO. Figure 3b depicts M_Fe_ vs. H*_a_* curves with a tendency towards saturation from 0.5 and 1% samples giving a maximum value of M_Fe_ = 0.11 emu/g and 0.24 emu/g at H*_a_* = 30 kOe, respectively.

The ZFC M(H*_a_*) loops also revealed first-field-induced magnetic anisotropy, which can be quantified as a spontaneous exchange bias (EB) field $H_{EB}=\left([H_{C}^{+}\right]-[H_{C}^{-}])/2$, where $H_{C}^{+}$ and $H_{C}^{-}$ corresponds to coercivity in the first- and second-curve of the M(H*_a_*) loop at which the magnetization is zero [3,41]. Figure 3c compares the ZFC and FC M(H*_a_*) loops near zero-field, where the inset gives full M(H*_a_*) loops measured over ±30 kOe from 0.5% sample. The effect of the cooling-field leads to further enhancement in the magnetic anisotropy. Figure 3d gives a plot of H_EB_ obtained from both ZFC and FC M(H*_a_*) loops vs. Fe-concentration *x*, where the inset of the figure depicts the coercivity $H_{C}=\left(H_{C}^{+}-H_{C}^{-}\right)/2$ vs. *x*. Almost similar values of H_C_ were obtained from both ZFC and FC M(H*_a_*) loops, whereas an enhanced value of the EB field from FC M(H*_a_*) loops. A sudden enhancement in the values of coercivity H_C_ can be seen from a 0.5% Fe-doped NiO sample, reaching a maximum value of 809 Oe at 1% (inset of Figure 3d). A maximum value of H_EB_ = −316 Oe is obtained after FC M(H*_a_*) loop from NiO NPs, and its value decreases with the increase of *x*. Note that the obtained values from M(H*_a_*) loops are not intrinsic since saturated hysteresis is not achievable even with a maximum field of 50 kOe. Moreover, the obtained conventional and spontaneous EB field from NiO NPs is consistent with previous findings and it can be attributed to the formed uncompensated core and disordered shell-type structure [3,4]. The observed reduction in the EB field from Fe-doped NiO NPs and its further reduction with the increase of Fe-concentration could be understood by assuming the presence of a 4:1 defect cluster in the core of the NPs (inset of Figure 3b) [16]. The 4:1 defect cluster consists of tetravalent interstitial iron Fe_i_^4+^ and four V_Ni_, in total, being four times negatively charged. The formation of such defect clusters could result in the enhancement of V_Ni_ in the core of NPs and, consequently, suppression in AF anisotropy of host NiO [27]. Therefore, for a very weak AF anisotropy, one could only see an enhancement in the H_C_ without any EB field [42].

### 3.4. Temperature Dependence of Magnetization

**Figure 4:**
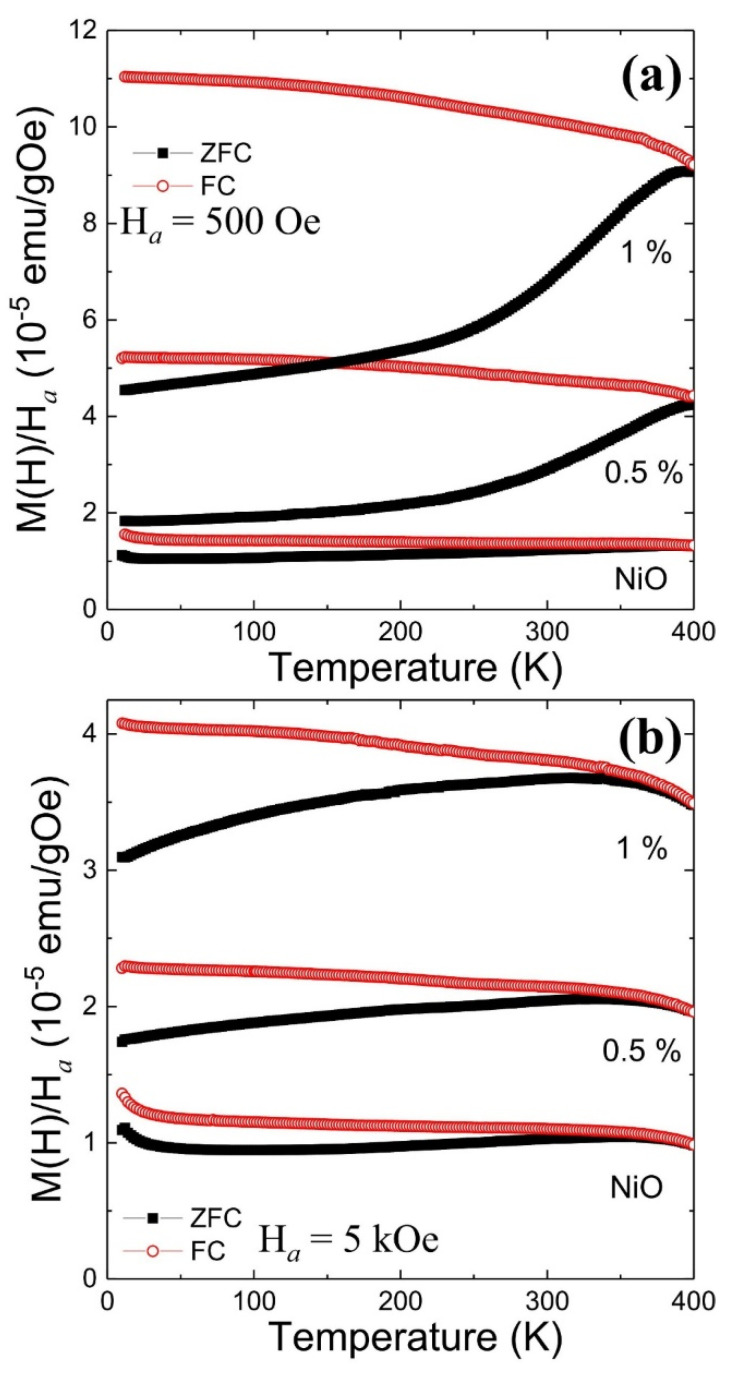
(**a**,**b**) M(H)/H*_a_* vs. T plots for NiO, 0.5, and 1% samples measured using ZFC and FC protocols at external fields H*_a_* of 500 Oe and 5 kOe, respectively.

The temperature-dependent magnetization measurements were carried out using ZFC and FC protocols. During ZFC measurement, the applied magnetic field was set to zero while using oscillator mode, and then the SQUID magnet was reset to remove any stray field at 400 K. Figure 4a,b show the M(H)/H*_a_* vs. T plots for NiO, 0.5%, and 1% samples at external fields H*_a_* of 500 Oe and 5 kOe, respectively. An increase in the magnetization with Fe-concentration can be seen consistent with the NPD and M(H*_a_*). At 500 Oe, the ZFC-FC curves of all samples remain bifurcated, even up to 400 K (defined as irreversible temperature *T_irr_* where $\left(M_{FC}-M_{ZFC}\right)=0$), suggesting blocking temperature *T_B_* (ZFC maximum) lying above the measured temperature range. With the increase of the external magnetic field, a relatively broadened ZFC curve appears from Fe-doped NiO NPs as compared to pure NiO, and its maximum shifts towards lower temperatures. The obtained *T_B_* at 5 kOe field from NiO, 0.5%, and 1% sample is around 350, 334, and 316 K, respectively. Whereas, the *T_irr_*, which can be considered as the onset temperature of the freezing process for NiO, 0.5%, and 1% sample, lies above 400 K. Assuming the assemblies of non-interacting NPs, the relaxation of magnetization with a uniaxial magnetic anisotropy can be described by the Néel–Arrhenius law: $K\left(x\right)=25k_{B}T_{B}\left(x\right)/V\left(x\right)$, where K(x) is the magnetocrystalline anisotropy of NPs, k_B_ is Boltzmann constant, and *V(x)* is the median of the particle volume distribution. The calculated value of K from NiO, 0.5% and 1% samples at 5 kOe is $0.9232\times 10^{4}\;\mathrm{erg}/{\mathrm{cm}}^{3}$, $1.020\times 10^{4}\;\mathrm{erg}/{\mathrm{cm}}^{3}$, and $1.400\times 10^{4}\;\mathrm{erg}/{\mathrm{cm}}^{3}$, respectively. The obtained value of K from NiO NPs is much smaller than that of value from bulk $K_{\mathrm{AF}}\left(0\right)=4.96\times 10^{6}\;\mathrm{erg}/{\mathrm{cm}}^{3}$ [43]. The increase in the value of K with the Fe-concentration could be a consequence of the decrease of particle size.

Irrespective of the external magnetic field, a sudden increase in the magnetization can be seen in the low-temperature region from ZFC-FC curves of NiO NPs, which could be related to the collective freezing of disordered surface spins. On the other hand, the response of ZFC-FC curves from 0.5% and 1% samples varies with an external magnetic field. At 500 Oe, a plateau is observed below 100 K from the FC curve of 0.5%, and 1% samples. Such FC curve shape in the low-temperature region is usually observed for super-spin-glasses (SSG) and super-ferromagnetic (SFM) materials, suggesting collective spin behavior [44]. At a finite interparticle distance in the magnetic system, more complex systems can be encountered due to magnetic interactions, especially of dipolar origin. Such a collective spin behavior in the magnetic system evolves with the increase of the interaction strength, modified superparamagnetic (SPM) behavior, SSG, and SFM states [45]. Therefore, in the present system, due to the presence of a 4:1 defect cluster in the core of the NPs and the enhanced magnetization, the interparticle and intraparticle interactions at a finite distance could have enhanced further with the increase of Fe-doping concentration. At 5 kOe, a saturation like behavior can be seen from the FC curve, whereas a huge broadening in the ZFC curve associated with the large distribution of energy barriers. The above findings suggest the existence of two almost independent contributions to the measured M(H*_a_*) and M(T) curves: one reflecting the antiferromagnetism of NiO (linear in H and weakly temperature-dependent). Another reflecting the NP size distribution and weak ferromagnetic (excess) magnetic moment with particle anisotropies yielding relaxation time according to Arrhenius dynamics and M vs. H*_a_* curves governed by Langevin functions.

### 3.5. Time Dependence of Magnetization

**Figure 5:**
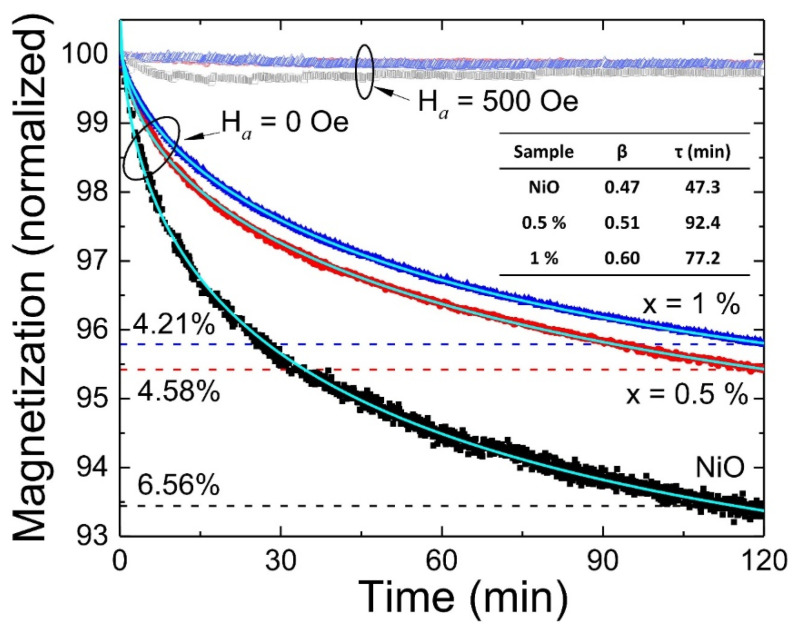
A plot of normalized magnetic moment relaxation measured concerning time M(t) at 300 K for 2 h from NiO (black), 0.5% (Red) and 1% (blue) samples in an applied field H*_a_* = 500 Oe (light color) and 0 Oe (dark color). The solid line represents a satisfactory linear fit to M(t) curves measured in 0 Oe using stretched exponential function with fitted parameters, as depicted in the inset. The horizontal dashed lines correspond to the amount of relaxation for 2 h in zero applied field for each composition.

Time dependency of the magnetization relaxation M(t) measurement was carried out both with and without the external magnetic field in order to investigate the effect of Fe-doping concentration on the magnetic anisotropy energy barriers. Typically, the sample was initially cool down from 400 K to 300 K in an applied field of 500 Oe, and the M(t) curves were recorded for a duration of 2 h at zero and 500 Oe fields. The magnetic field was set to zero while using oscillator mode, and then the SQUID magnet was reset to remove any stray field at 300 K. Figure 5 compares the normalized time-dependent magnetic moment relaxation obtained in 0 and 500 Oe from NiO, 0.5% and 1% samples. Very weak relaxation in the magnetic moment was observed from the M(t) curves measured at 500 Oe field. Whereas, a broad relaxation with about 6.56% drop in the magnetization was obtained from NiO NPs at 0 Oe field. The observed broad relaxation is governed by distribution in the particle size as well as exchange coupling anisotropy. Interestingly, with the increase of Fe-doping concentration from 0.5% to 1%, relaxation broadened, and the amount of magnetization dropped further from 4.58% to 4.21%. The above findings suggest the effect of 1% Fe-doping has slow down the magnetic moment relaxation by around 35.8% concerning NiO at 300 K over 2 h. Because, firstly, the mean diameter distribution obtained from SEM images has shown a very similar size distribution for NiO, 0.5, and 1% samples, and, secondly, the exchange coupling anisotropy has reduced further with the increase of Fe-doping concentration. Hence, apart from size distribution, the intraparticle interactions due to formed 4:1 defect clusters may have created additional magnetic anisotropy energy barriers affecting the overall magnetic moment relaxation process. In this context, a well-known stretched exponential function was successfully used elsewhere to describe the effect of distribution in anisotropy energy barriers on the process of magnetic moment relaxation [46]. The solid lines in Figure 5 represent a satisfactory fit using a stretched exponential function $M\left(t\right)=m_{o}-m_{e}exp\left(-{\left(t/\tau \right)}^{\beta }\right)$ and the fitted value of τ and *β* are depicted. *m_o_* is an intrinsic magnetic component, *m_e_* glassy component, τ characteristic relaxation time, and *β* is stretching parameter. *m_e_* and τ are the function of measuring temperature and time, whereas *β* (0<β≤1) is a function of the measuring temperature only. In the above expression, depending on the value of *β*, the system either relaxes with a single time constant (β=1), or it involves activation against multi magnetic anisotropy energy barriers (β<1). The fitted value of *β* obtained from NiO, 0.5, and 1% samples is 0.47, 0.51, and 0.60, respectively, which suggests activation against multiple anisotropy energy barriers from both undoped and Fe-doped NiO NPs. Therefore, along with particle size distribution existence of multiple anisotropy energy barriers possibly bears a correlation to the signature of the presence of intraparticle interactions in Fe-doped NiO NPs.

### 3.6. Magnetic Memory Effect

**Figure 6:**
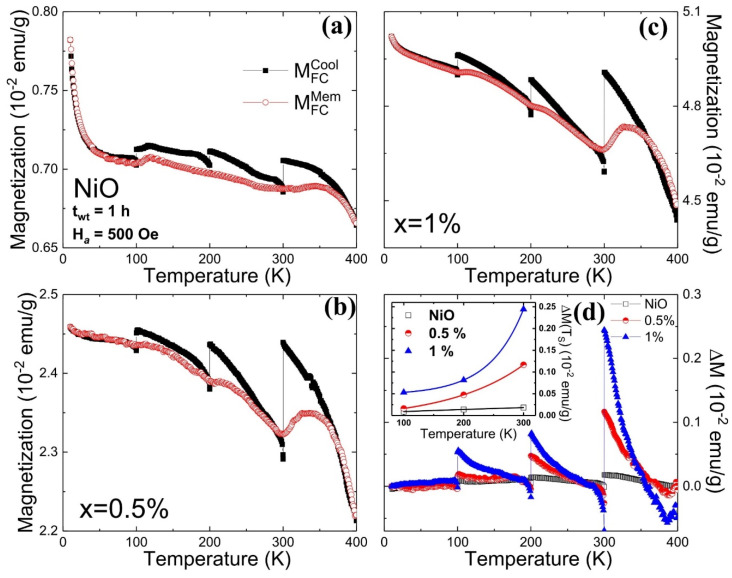
The FC magnetic memory effect from (**a**) NiO, (**b**) 0.5%, and (**c**) 1% samples. (**d**) The difference in magnetization $∆M=M_{\mathrm{FC}}^{\mathrm{cool}}-M_{\mathrm{FC}}^{\mathrm{mem}}$ concerning temperature. The value of maximum $∆M\left(T_{S}\right)$ at each stopping temperature, T_S_ is given in the inset of a figure (**d**).

Both FC and ZFC protocols suggested by Sun et al. were used to study the magnetic memory effect for possible future applications [47]. FC magnetic memory test: sample was first cooled down in an applied field H*_a_* = 500 Oe from 400 K, where sporadic stops for t_wt_ = 1 h in 0 Oe were given at various stopping temperatures *T_S_* (300 K, 200 K, and 100 K, below T_B_); above curve is designated as $M_{\mathrm{FC}}^{\mathrm{Cool}}$. Subsequently, magnetization was recorded while warming in the same applied field and the curve is designated as $M_{\mathrm{FC}}^{\mathrm{Mem}}$. Figure 6a–c depict the obtained FC memory effect results from NiO, 0.5%, and 1% samples, respectively. The amount of recovery of spins depends upon how fast the NPs realign to the applied magnetic field and, therefore, it can be quantified as $∆M\left(T_{S}\right)=M_{\mathrm{FC}}^{\mathrm{cool}}-M_{\mathrm{FC}}^{\mathrm{mem}}$ (Figure 6d). The value of $∆M\left(T_{S}\right)$ at each intermittent stopping temperature, *T_S_* reaches to its maximum and shows an increasing trend with the increase of temperature. An enhanced value of $∆M\left(T_{S}\right)$ is obtained from a 0.5% sample, and its value increases further with the increase of Fe-concentration to 1% (inset of Figure 6d). The above findings suggest that the step-like time dynamic magnetization measurement is reproduced upon warming from Fe-doped NiO NPs at RT. On the other hand, strongly exchange-coupled NiO NPs do not retain any sign of memory effect. Furthermore, an increasing behavior of $M_{\mathrm{FC}}^{\mathrm{Cool}}$ with the decrease of temperature can be seen from Fe-doped NiO NPs. Such type of behavior is commonly assigned to a non-interacting SPM system [48]. Contrary to it, an interacting spin-glass (SG) system shows a decrease of $M_{\mathrm{FC}}^{\mathrm{Cool}}$ with a decrease in temperature [49]. The SG system can be identified by measuring the ZFC memory effect. ZFC magnetic memory test: the sample was first ZFC from 400 K to 10 K, and then magnetization was recorded during heating in an applied field of 500 Oe; this curve is designated as a reference curve. The sample was again ZFC from 400 K, but now with a stop-and-wait protocol at 200 K and 100 K, where the sample is aged for 1 h duration, and then again cooled down to 10 K. Subsequently, magnetization was recorded, as done in the reference curve; this curve is designated as wait curve. The field was set to zero using the inbuild oscillatory mode, and then the stray field was removed by resetting the SQUID magnet. Usually, the difference between the wait and reference curve is characterized by a dip at the waiting temperature [48,49]. However, no dip was seen from both NiO and Fe-doped NiO samples (Figure S3).

**Figure 7:**
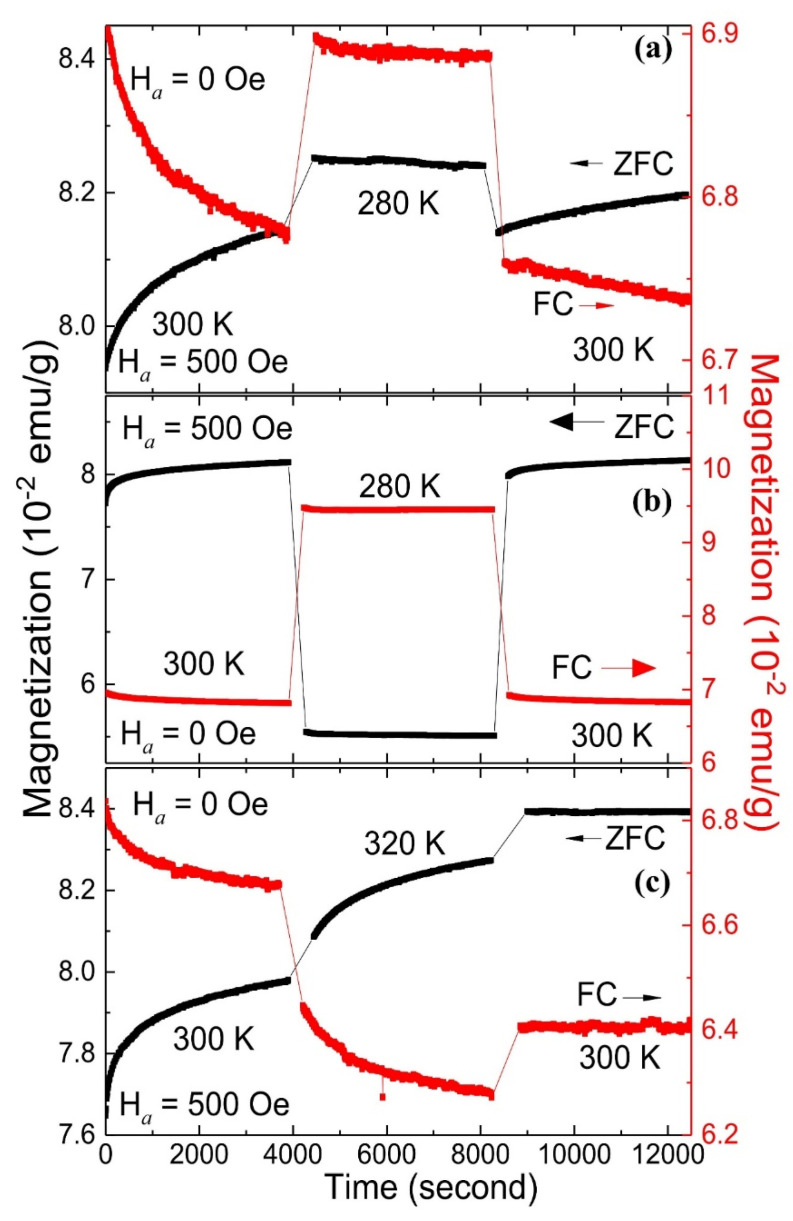
The effect of temperature-cooling (**a**) without (**b**) with field switching and (**c**) temperature-heating cycle without field switching during ZFC and FC magnetization relaxation from 1% sample.

The RT memory effect was further investigated by studying the effect of both the temperature-cooling and -heating cycle and the field switching with ZFC and FC magnetization relaxation protocols in a 1% sample (Figure 7) [47]. In ZFC (FC) relaxation magnetization measurement, sample was initially cooled from 400 to 300 K under zero-field (500 Oe) and the magnetization M(t) was measured for t_1_ = 4000 s at 500 Oe field (zero fields); after that, the sample was cooled to 280 K in the same magnetic field and magnetization was measured over time t_2_ = 4000 s. Finally, the sample was warmed back to 300 K and magnetization was measured for t_3_ = 4000 s. The relaxation in magnetization returns to the previous level, even after a temporary period that the sample was cooled to 280 K (Figure 7a). A similar phenomenon is also observed from ZFC and FC magnetization relaxation when the magnetic field was switched to 0 Oe and 500 Oe during t_2_ with the temporary cooling at 280 K, respectively (Figure 7b). The above measurement shows that magnetization returns to the previous level when the temperature and magnetic field are returned to the previous condition at 300 K and 500 Oe. However, both ZFC and FC relaxation magnetization does not restore its previous state before the temporary heating to 320 K, demonstrating no memory effect (Figure 7c).

## 4. Discussion and Conclusions

The observed asymmetric response concerning positive and negative temperature changes is following a hierarchical model for an interacting particle system [50,51]. The above model is also applicable to the non-interacting SPM system [32] and the exchange-coupled system [33], according to recent findings. In the former case, the distribution in energy barriers originated from the particle size distribution, whereas, in the latter case, from interface exchange-coupling. However, in the studied system, one could see that the effect of the cooling field has led to an enhancement in the exchange bias field both in the pure NiO and Fe-doped NiO NPs. The observed decrease in the EB field with the increase of Fe-doping concentration and the absence of memory effect in strongly exchange-coupled NiO NPs (having size distribution, i.e., anisotropy distribution) suggested RT memory effect and EB phenomenon are independent. Hence, it appears that, along with particles uniaxial anisotropy [17], the intraparticle interactions due to formed 4:1 defect clusters [16,23] may have paved the way for creating an additional anisotropy energy barrier, resulting in an appearance of RT memory effect from Fe-doped NiO NPs. In conclusion, Ni_1-x_Fe_x_O NPs with x(%) varying from 0 to 1% and having a similar structure as that of NiO without any impurity phase was successfully synthesized while using the co-precipitation method. The effect of Fe-doping from 0 to 1% leads to a decrease of particle size from 63 nm to 53 nm and an increment of the magnetic moment of 3*d* ions (evident from NPD experiment). The RT ferromagnetic properties and magnetic memory effect is accomplished in Fe-doped NiO NPs studied by different protocols of ZFC and FC magnetization relaxation measurements. Our findings suggested that, as compared to non-interacting or interacting SPM NPs and exchange-coupled systems, the intrinsic intraparticle interaction in the Fe-doped NiO system has created an additional anisotropy energy barrier that can be tailored simply by controlling Fe-dopant concentration. The outcome of this is technologically attractive for the future development of RT ferromagnetism in AF NiO in order to facilitate the possible integration of spintronic devices.

## Supplementary Materials

The following are available online at https://www.mdpi.com/2079-4991/10/7/1318/s1, Figure S1: Rietveld refined (red line) PXRD spectra (dots) from Ni_1-x_Fe_x_O NPs, Figure S2: M(H*_a_*) loops measured from 5% and 10% samples at 300 K, Figure S3: ZFC magnetic memory effect: Difference between the wait and reference curve from (**a**) NiO, (**b**) 0.5%, and 1% samples. Table S1: Rietveld refined parameters obtained from PXRD spectra in cubic *Fm*$\bar{3}m$ phase. Table S2: Rietveld refined parameters obtained from NPD spectra in cubic *Fm*$\bar{3}m$ phase. Table S3: Rietveld refined parameters obtained from NPD spectra in rhombohedral *R*$\bar{3}m$ phase.

## References

[1] Kodama R.H., Makhlouf S.A., Berkowitz A.E. (1997). Finite Size Effects in Antiferromagnetic NiO Nanoparticles. Phys. Rev. Lett..

[2] Gandhi A.C., Wu S.Y. (2017). Strong Deep-Level-Emission Photoluminescence in NiO Nanoparticles. Nanomaterials.

[3] Gandhi A.C., Pant J., Wu S.Y. (2016). Dense inter-particle interaction mediated spontaneous exchange bias in NiO nanoparticles. RSC Adv..

[4] Mandal S., Banerjee S., Menon K.S.R. (2009). Core-shell model of the vacancy concentration and magnetic behaviour for antiferromagnetic nanoparticle. Phys. Rev. B.

[5] Gandhi A.C., Pant J., Pandit S.D., Dalimbkar S.K., Chan T.S., Cheng C.L., Ma Y.R., Wu S.Y. (2013). Short-Range Magnon Excitation in NiO Nanoparticles. J. Phys. Chem. C.

[6] Rinaldi-Montes N., Gorria P., Martínez-Blanco D., Fuertes A.B., Fernández Barquín L., Rodríguez Fernández J., de Pedro I., Fdez-Gubieda M.L., Alonso J., Olivi L. (2014). Interplay between microstructure and magnetism in NiO nanoparticles: Breakdown of the antiferromagnetic order. Nanoscale.

[7] Tiwari S.D., Rajeev K.P. (2005). Signatures of spin-glass freezing in NiO nanoparticles. Phys. Rev. B.

[8] Lee D.U., Fu J., Park M.G., Liu H., Kashkooli A.G., Chen Z. (2016). Self-Assembled NiO/Ni(OH)_2_ Nanoflakes as Active Material for High-Power and High-Energy Hybrid Rechargeable Battery. Nano Lett..

[9] Rinaldi-Montes N., Gorria P., Martínez-Blanco D., Fuertes A.B., Puente-Orench I., Olivi L., Blanco J.A. (2016). Size effects on the Néel temperature of antiferromagnetic NiO nanoparticles. AIP Adv..

[10] Oka K., Yanagida T., Nagashima K., Kawai T., Kim J.S., Park B.H. (2010). Resistive-Switching Memory Effects of NiO Nanowire/Metal Junctions. J. Am. Chem. Soc..

[11] Sasaki T., Devred F., Eloy P., Gaigneaux E.M., Hara T., Shimazu S., Ichikuni N. (2009). Development of supported NiO nanocluster for aerobic oxidation of 1-Phenylethanol and elucidation of reaction mechanism via X-ray analysis. Bull. Chem. Soc. Jpn..

[12] Ouyang Y., Huang R., Xia X., Ye H., Jiao X., Wang L., Lei W., Hao Q. (2018). Hierarchical structure electrodes of NiO ultrathin nanosheets anchored to NiCo_2_O_4_ on carbon cloth with excellent cycle stability for asymmetric supercapacitors. Chem. Eng..

[13] Wang J., Cai J., Lin Y.-H., Nan C.-W. (2005). Room-temperature ferromagnetism observed in Fe-doped NiO. Appl. Phys. Lett..

[14] Shim J.H., Hwang T., Lee S., Park J.H., Han S.J., Jeong Y.H. (2005). Origin of ferromagnetism in Fe- and Cu-codoped ZnO. Appl. Phys. Lett..

[15] Lee H.-J., Jeong S.-Y., Cho C.R., Park C.H. (2002). Study of diluted magnetic semiconductor: Co-doped ZnO. Appl. Phys. Lett..

[16] Gandhi A.C., Pradeep R., Yeh Y.C., Li T.Y., Wang C.Y., Hayakawa Y., Wu S.Y. (2019). Understanding the Magnetic Memory Effect in Fe-Doped NiO Nanoparticles for the Development of Spintronic Devices. ACS Appl. Nano Mater..

[17] Moura K.O., Lima R.J.S., Coelho A.A., Souza-Junior E.A., Duque J.G.S., Meneses C.T. (2014). Tuning the surface anisotropy in Fe-doped NiO nanoparticles. Nanoscale.

[18] Kurokawa A., Sakai N., Zhu L., Takeuchi H., Yano S., Yanoh T., Onuma K., Kondo T., Miike K., Miyasaka T. (2013). Magnetic properties of Fe-doped NiO nanoparticles. J. Korean Phys. Soc..

[19] Mishra A.K., Bandyopadhyay S., Das D. (2012). Structural and magnetic properties of pristine and Fe-doped NiO nanoparticles synthesized by the co-precipitation method. Mater. Res. Bull..

[20] Mallick P., Rath C., Biswal R., Mishra N.C. (2009). Structural and magnetic properties of Fe doped NiO. Indian J. Phys..

[21] Manna S., Deb A.K., Jagannath J., De S.K. (2008). Synthesis and Room Temperature Ferromagnetism in Fe Doped NiO Nanorods. J. Phys. Chem. C.

[22] He J.H., Yuan S.L., Yin Y.S., Tian Z.M., Li P., Wang Y.Q., Liu K.L., Wang C.H. (2008). Exchange bias and the origin of room-temperature ferromagnetism in Fe-doped NiO bulk samples. J. Appl. Phys..

[23] Grimes R.W., Anderson A.B., Heuer A.H. (1987). Interaction of dopant cations with 4:1 defect clusters in non-stoichiometric 3d transition metal monoxides: A theoretical study. J. Phys. Chem. Solids.

[24] Krafft K.N., Martin M. (1998). A Thermogravimetric Study of the Non-Stoichiometry of Iron-Doped Nickel Oxide (Ni_1−x_Fe_x_)_1−δ_O. Korean J. Ceram..

[25] Hoser A., Martin M., Schweika W., Carlsson A.E., Caudron R., Pyka N. (1994). Diffuse neutron scattering of iron-doped nickel oxide. Solid State Ion..

[26] Haaß F., Buhrmester T., Martin M. (2001). High-temperature in situ X-ray absorption studies on the iron valence in iron-doped nickel oxide (Ni_1−x_Fe_x_)_1−δ_O. Solid State Ion..

[27] Haaß F., Buhrmester T., Martin M. (2001). Quantitative elaboration of the defect structure of iron doped nickel oxide (Ni_0.955_Fe_0.045_)_1−δ_O by in situ X-ray absorption spectroscopy. Phys. Chem. Chem. Phys..

[28] Gandhi A.C., Chan T.S., Pant J., Wu S.Y. (2017). Strong Pinned-Spin-Mediated Memory Effect in NiO Nanoparticles. Nanoscale Res. Lett..

[29] Bisht V., Rajeev K.P. (2009). Memory and aging effects in NiO nanoparticles. J. Phys. Condens. Matt..

[30] Xu L., Gao Y., Malik A., Liu Y., Gong G., Wang Y., Tian Z., Yuan S. (2019). Field pulse induced magnetic memory effect at room temperature in exchange coupled NiFe_2_O_4_/NiO nanocomposites. J. Mag. Mag. Mater..

[31] Chakraverty S., Ghosh B., Kumar S., Frydman A. (2006). Magnetic coding in systems of nanomagnetic particles. Appl. Phys. Lett..

[32] Tsoi G.M., Senaratne U., Tackett R.J., Buc E.C., Naik R. (2005). Memory effects and magnetic interactions in a γ-Fe_2_O_3_ nanoparticle system. J. Appl. Phys..

[33] Tian Z., Xu L., Gao Y., Yuan S., Xia Z. (2017). Magnetic memory effect at room temperature in exchange coupled NiFe_2_O_4_-NiO nanogranular system. Appl. Phys. Lett..

[34] Dhara S., Chowdhury R.R., Bandyopadhyay B. (2015). Strong memory effect at room temperature in nanostructured granular alloy Co_0.3_Cu_0.7_. RSC Adv..

[35] Balagurov A.M., Bobrikov I.A., Sumnikov S.V., Yushankhai V.Y., Grabis J., Kuzmin A., Mironova-Ulmane N., Sildos I. (2016). Neutron diffraction study of microstructural and magnetic effects in fine particle NiO powders. Phys. Status Solidi.

[36] Brok E., Lefmann K., Deen P.P., Lebech B., Jacobsen H., Nilsen G.J., Keller L., Frandsen C. (2015). Polarized neutron powder diffraction studies of antiferromagnetic order in bulk and nanoparticle NiO. Phys. Rev. B.

[37] Roth W.L. (1958). Magnetic Structures of MnO, FeO, CoO, and NiO. Phys. Rev..

[38] Lisa T., Matthew G.T., David A.K., Peter M.M.T., Paul J.S., Andrew L.G. (2016). Exploration of antiferromagnetic CoO and NiO using reverse Monte Carlo total neutron scattering refinements. Phys. Scr..

[39] Rinaldi-Montes N., Gorria P., Martínez-Blanco D., Fuertes A.B., Fernández Barquín L., Rodríguez Fernández J., de Pedro I., Fdez-Gubieda M.L., Alonso J., Olivi L. (2015). On the exchange bias effect in NiO nanoparticles with a core(antiferromagnetic)/shell (spin glass) morphology. J. Phys. Conf. Ser..

[40] Cooper J.F.K., Ionescu A., Langford R.M., Ziebeck K.R.A., Barnes C.H.W., Gruar R., Tighe C., Darr J.A., Thanh N.T.K., Ouladdiaf B. (2013). Core/shell magnetism in NiO nanoparticles. J. Appl. Phys..

[41] Magnan H., Bezencenet O., Stanescu D., Belkhou R., Barbier A. (2010). Beyond the magnetic domain matching in magnetic exchange coupling. Phys. Rev. Lett..

[42] Nogués J., Sort J., Langlais V., Skumryev V., Suriñach S., Muñoz J.S., Baró M.D. (2005). Exchange bias in nanostructures. Phys. Rep..

[43] Kondoh H. (1960). Antiferromagnetic resonance in NiO in far-infrared region. J. Phys. Soc. Jpn..

[44] Perzanowski M., Marszalek M., Zarzycki A., Krupinski M., Dziedzic A., Zabila Y. (2016). Influence of Superparamagnetism on Exchange Anisotropy at CoO/[Co/Pd] Interfaces. ACS Appl. Mater. Interfaces.

[45] Chen X., Bedanta S., Petracic O., Kleemann W., Sahoo S., Cardoso S., Freitas P.P. (2005). Superparamagnetism versus superspin glass behaviour in dilute magnetic nanoparticle systems. Phys. Rev. B.

[46] Khan N., Mandal P., Prabhakaran D. (2014). Memory effects and magnetic relaxation in single-crystalline La_0.9_Sr_0.1_CoO_3_. Phys. Rev. B.

[47] Sun Y., Salamon M.B., Garnier K., Averback R.S. (2003). Memory effects in an interacting magnetic nanoparticle system. Phys. Rev. Lett..

[48] Sasaki M., Jönsson P.E., Takayama H., Mamiya H. (2005). Aging and memory effects in superparamagnets and superspin glasses. Phys. Rev. B.

[49] Bandyopadhyay M., Dattagupta S. (2006). Memory in nanomagnetic systems: Superparamagnetism versus spin-glass behaviour. Phys. Rev. B.

[50] Parisi G. (1979). Infinite number of order parameters for spin-glasses. Phys. Rev. Lett..

[51] Parisi G. (1983). Order Parameter for Spin-Glasses. Phys. Rev. Lett..

