# Supplementary material for: Room Temperature Magnetic Memory Effect in Cluster-Glassy Fe-Doped NiO Nanoparticles
Source: Nanomaterials (Basel). 2020 Jul 4;10(7):1318. doi: 10.3390/nano10071318 (PMC7407623; doi:10.3390/nano10071318)
Supplement: Supplementary file 1 [file nanomaterials-10-01318-s001.pdf]

Supplementary information

Room temperature magnetic memory effect in cluster-glassy Fe-doped NiO nanoparticles

Ashish Chhaganlal Gandhi,[1] Tai-Yue Li,[1] B. Vijaya Kumar,[2] P. Muralidhar Reddy [2] Jen-Chih Peng,[3] Chun-Ming Wu,[3] and Sheng Yun Wu[*1]

[1]Department of Physics, National Dong Hwa University, Hualien 97401, Taiwan.

[2]Department of Chemistry, Nizam College, Osmania University, Hyderabad-500001, India.

[3]Synchrotron radiation research center, Hsinchu 30076, Taiwan

*Correspondence to sywu@mail.ndhu.edu.tw (SYW)

Figure S1. Rietveld refined (red line) PXRD spectra (dots) from $Ni_{1-x}Fe_xO$ NPs with x = 0, 0.5, 1, 5 and 10 % (bottom to top). The corresponding fitting parameters are given in Table S1.

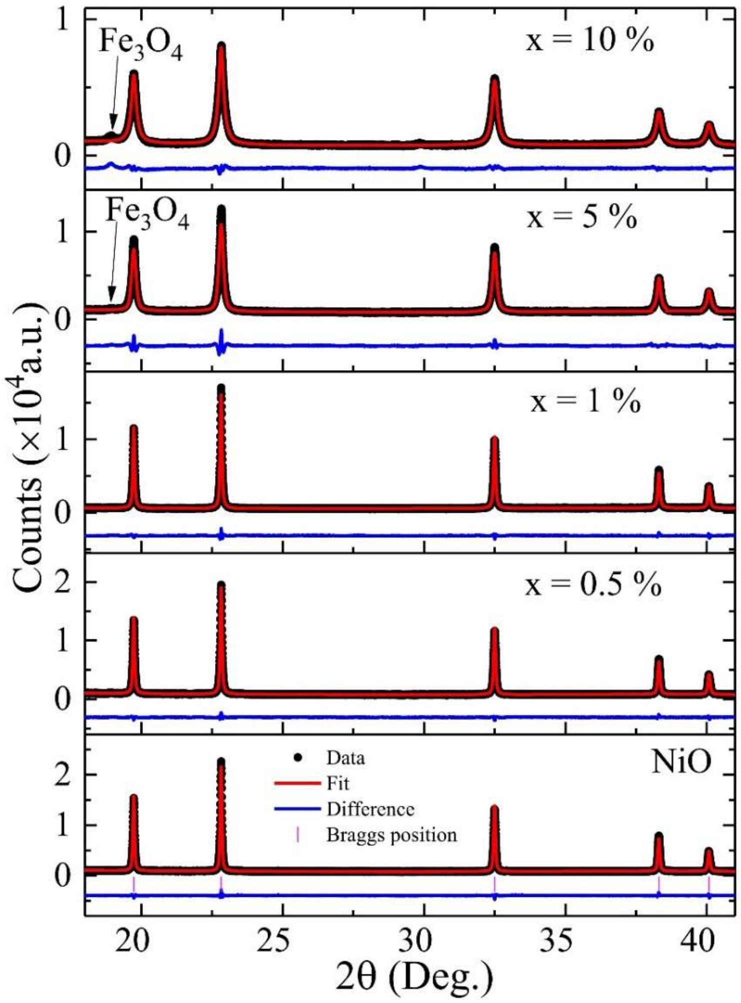

Figure S2. M(H_a) loops measured from 5 % and 10 % samples at 300 K.

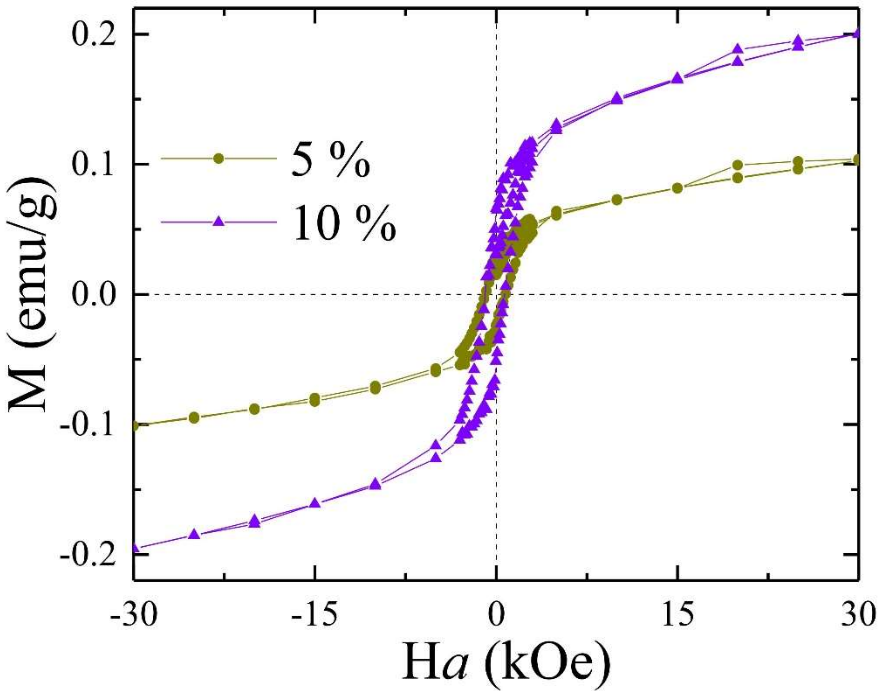

Figure S3. ZFC magnetic memory effect: Difference between the wait and reference curve from (a) NiO, (b) 0.5 %, and 1 % samples.

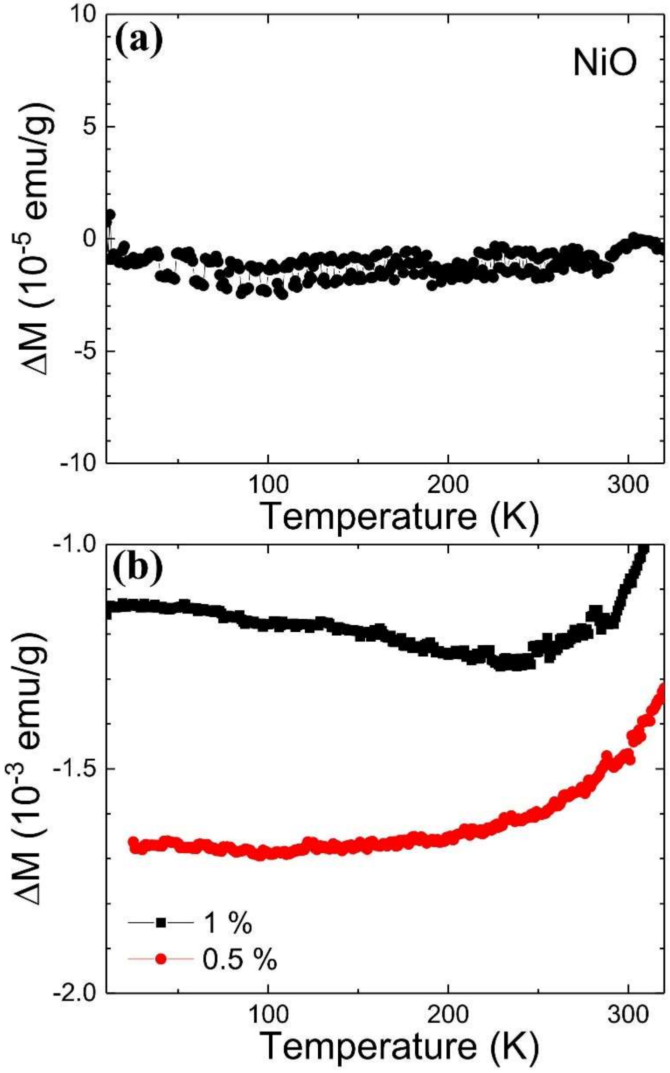

Table S1. Rietveld refined parameters (lattice constant, R-factors) obtained from PXRD spectra in cubic $Fm\bar{3}m$ phase. Calculated grain size $d_{(200)}$ using Scherrer formula.

Sample	a (Å)	ρ_{nuc} (g/cm^3)	Rp	Rwp	Re	χ^2	Bragg R-Factor	Rf-Factor	$d_{(200)}$ (nm)
NiO	4.17766(3)	6.848	5.54	5.61	3.59	2.45	1.08	0.514	68.3
0.5 %	4.17729(3)	6.852	5.30	5.26	3.82	1.90	0.848	0.408	64.2
1 %	4.17733(3)	6.864	5.17	5.55	3.68	2.27	0.807	0.514	67.9
5 %	4.17714(9)	6.898	10.3	10.8	3.88	7.79	2.15	1.48	31.2
10 %	4.17708(8)	6.887	8.80	9.57	5.64	2.88	1.94	0.934	20.8

Table S2. Rietveld refined parameters obtained from NPD spectra in cubic $Fm\bar{3}m$ phase.

Sample	Rp	Rwp	Re	X^2	R_B	R_F	R_M	*a* (Å)	U	-V	W	ρ_{nuc} (g/cm^3)	ρ_{mag} (g/cm^3)	$m*\mu_B$
300 K														
NiO	8.59	9.06	3.86	5.53	0.552	0.251	4.53	4.1783(2)	2.99(20)	1.49(35)	1.08(14)	6.802	1.336	1.232(18)
0.5 %	7.93	8.41	3.82	4.85	0.440	0.203	4.50	4.1807(2)	3.11(19)	1.75(33)	1.20(13)	6.790	1.334	1.305(17)
1 %	8.19	8.48	3.71	5.23	0.698	0.442	5.94	4.1802(2)	3.15(21)	1.74(35)	1.17(14)	6.793	1.334	1.318(19)
10 K														
NiO	9.09	9.34	3.82	5.99	0.803	0.377	6.76	4.1752(2)	3.04(20)	1.63(34)	1.16(14)	6.817	1.339	1.258(18)
0.5 %	8.50	8.75	3.78	5.37	0.578	0.338	5.56	4.1766(2)	3.05(19)	1.52(33)	1.11(13)	6.810	1.338	1.340(18)
1 %	7.92	8.53	3.68	5.37	0.472	0.273	7.15	4.1744(2)	3.32(20)	1.99(34)	1.28(14)	6.821	1.340	1.339(17)

Table S3. Rietveld refined parameters obtained from NPD spectra in rhombohedral $R\bar{3}m$ phase.

Sample	Rp	Rwp	Re	X^2	R_B	R_F	R_M	*a* (Å)	c (Å)	-U	V	-W	ρ_{nuc} (g/cm^3)	ρ_{mag} (g/cm^3)	m*μ_B
300 K															
NiO	9.32	10.1	3.88	6.73	1.31	1.39	64.5	2.9556(2)	7.1581(7)	0.94(22)	3.03(36)	0.14(14)	6.872	1.800	1.636(30)
0.5 %	7.58	8.40	3.83	4.81	1.01	0.724	18.2	2.9630(2)	7.1771(6)	0.56(19)	1.98(31)	0.21(13)	6.819	1.786	1.837(23)
1 %	9.21	10.5	3.69	8.17	1.56	1.24	35.3	2.9625(2)	7.1744(7)	0.43(26)	1.55(44)	0.32(18)	6.825	1.788	1.840(29)
10 K															
NiO	7.58	8.60	3.81	5.09	0.767	0.480	12.3	2.9637(2)	7.1773(5)	0.64(18)	2.24(30)	0.13(12)	6.816	1.785	1.831(23)
0.5 %	7.12	8.31	3.78	4.84	0.861	0.644	19.1	2.9624(2)	7.1743(5)	0.67(18)	2.19(29)	0.12(12)	6.825	1.788	1.901(23)
1 %	7.10	8.28	3.68	5.05	0.867	0.591	17.3	2.9601(2)	7.1690(6)	0.47(19)	1.92(31)	0.22(12)	6.841	1.792	1.887(22)
